# Human Development VII: A Spiral Fractal Model of Fine Structure of Physical Energy Could Explain Central Aspects of Biological Information, Biological Organization and Biological Creativity

**DOI:** 10.1100/tsw.2006.256

**Published:** 2006-11-14

**Authors:** Søren Ventegodt, Tyge Dahl Hermansen, Trine Flensborg-Madsen, Erik Rald, Maj Lyck Nielsen, Joav Merrick

**Affiliations:** ^1^ Quality of Life Research Center, Teglgårdstræde 4-8, DK-1452 Copenhagen K, Denmark; ^2^ Research Clinic for Holistic Medicine, Copenhagen, Denmark; ^3^ Nordic School of Holistic Medicine, Copenhagen, Denmark; ^4^ Scandinavian Foundation for Holistic Medicine, Sandvika, Norway; ^5^ Zusman Child Development Center, Soroka University Medical Center, Ben Gurion University of the Negev, Beer-Sheva, Israel; ^6^ National Institute of Child Health and Human Development, Division for Mental Retardation, Ministry of Social Affairs, Jerusalem, Israel; ^7^ Office of the Medical Director, Division for Mental Retardation, Ministry of Social Affairs, Jerusalem, Israel

**Keywords:** holistic biology, theoretical biology, clinical holistic medicine, morphogenesis, ontogenesis, developmental biology, Denmark

## Abstract

In this paper we have made a draft of a physical fractal essence of the universe, a sketch of a new cosmology, which we believe to lay at the root of our new holistic biological paradigm. We present the fractal roomy spiraled structures and the energy-rich dancing “infinite strings” or lines of the universe that our hypothesis is based upon. The geometric language of this cosmology is symbolic and both pre-mathematical and pre-philosophical. The symbols are both text and figures, and using these we step by step explain the new model that at least to some extent is able to explain the complex informational system behind morphogenesis, ontogenesis, regeneration and healing. We suggest that it is from this highly dynamic spiraled structure that organization of cells, organs, and the wholeness of the human being including consciousness emerge. The model of “dancing fractal spirals” carries many similarities to premodern cultures descriptions of the energy of the life and universe. Examples are the Native American shamanistic descriptions of their perception of energy and the old Indian Yogis descriptions of the life-energy within the body and outside. Similar ideas of energy and matter are found in the modern superstring theories. The model of the informational system of the organism gives new meaning to Bateson 's definition of information: “A difference that makes a difference”, and indicates how information-directed self-organization can exist on high structural levels in living organisms, giving birth to their subjectivity and consciousness.

## INTRODUCTION

In previous papers we have seen how it is possible to put up a general model for information in biological systems. The model shows how exchange of information between elements on each level of the organism, and between these levels to a large extent can describe how information flows to give information-directed self-organization at all levels of the organism, and even of the supra-organismic level of the living realm. The problem is, as we also have seen, that we cannot explain this phenomenon with any known principle from either the chemical or the physical sciences. That gives us a great dilemma as what to believe. Either we hesitate and look for our mistake – and we have done that for years actually – or we step courageously forward to identify the new and undiscovered phenomenon – a new unseen and unknown structure of the universe - that allows life to carry, store, recall, and exchange information.

Let us for a moment consider what information is: “A difference that makes a difference” as Bateson wisely put it[1] can maybe give us a fine idea. How can this be done? Only if there is receptivity in the system, a key point where a very small energy can make a very large difference. We know this principle from the “Butterfly effect” in chaotic systems: the beat of a butterfly’s wing creates a cyclone in America. Most of us do not believe that it actually was the butterfly that did disturb the weather systems. The butterfly effect is an interesting phenomenon mostly met in the computer calculated world, where iterations allows a little lack of precision to be very disturbing a moment later. In life this is not seen either. You can almost stir the early embryo with a spoon without causing any noticeable difference. So living organisms are normally not perceptible for disturbances, but the question is: what is providing the information that gives all that structural stability?

Let us suggest that all the particles of a cell through many levels of interaction join into one large energetically, highly structured system, and that is what happens at the top level of this “global cell field” determines what happens everywhere in the cell – that means the cell is using “top down control”. What we need is to give a model for the structure of the collective field, presumably of quantum nature. It needs to be a model that can be visualized to make sense[2]. Highly complicated math will not help us much here, since we need something to see and understand.

This is therefore the effort of this paper: to present the first draft of such a fine structure of the physical energy. It should be able to control the local activities of the proteins through the delivery of a tiny energy that tips the balance between two states with a different profile of activity (cp. Bateson’s concept of information[1]); it should be able to store this *information*, it should be able to pack and carry it, to recall it and to exchange it with other cells in an organism, to create multi-cellular order.

### The Model Does Not Yet Exist

We were not able to find such a phenomenon described in natural scientist’s libraries. We found a lot of mathematical structures with fractals as the structure that looked the most “biological”, but they were often quite static and did not provide us with the dynamic properties needed for a structural informational backbone of a living cell. We (SV) went to the mathematicians at the University of Copenhagen, but they could not really help. Holger Bech Nielsen knew of some structures with a slight resemblance of what we needed (called Peano curves), but they were not dynamic in three dimensions. We ended up drawing hundreds of different geometrical patterns, until one day we come up with the spiral fractal, which we present as a first solution.

To some extent, our multidimensional spiral-fractal model seems to be able to explain the informational system for the organization of cells, organs and the whole being. It seems to be able to explain central aspects of both morphogenesis, ontogenesis, and consciousness of an individual. In addition to this we believe it is also able to explain important dimensions of evolution[3], the organization of higher biological levels, such as social activity in the family, communities, whole cultures and eco systems. It does not explain everything, it is not perfect, but we believe it is a start. So please take into consideration that what we present here is ongoing research, which from our side has been going on for nearly 20 year. The reason we present it now is that we really believe that we have found something important, although the issue in its complexity in many ways is too much of a mouthful for our small research institute. We definitely need a huge international cooperation to really face this challenge in a formal and strict scientific manner. But one reason to publish it was that similar structural descriptions of energy of life and energy of the universe has been collected by anthropologists interviewing shamans from many premodern cultures on their concepts of life-energy and universal energy[4,5,6]. Some modern experimental cosmologies also contain a concept of dynamic spirals[7].

What we found and what we continue to find surprising is that a simple geometrical "breaking up of the space-time" as the one we are proposing here, can "explain" complicated biological phenomena like enzymatic activity, cellular order, awareness, memory, and consciousness. The model we propose here is based on fractal spacious structures and bear many resemblances to the small energetic strings of modern physics (compare the superstrings[8]). We find it to some extent able to explain the distribution of information to organize the different levels and events of the morphogenesis. It is very good to explain the spontaneous self-organization of societies and ecosystems, and it can explain how the planet is one living organism (cp. The Gaia-hypothesis[9]) and the level of collective consciousness[4].

That is if you are willing to accept a "hint from a symbolic presentation" as an explanation. The reason why we accept that it makes so much sense and after all it allows us not only to explain the structure and functioning of the living, but also diseases and healing. It actually allows us to believe in very complex phenomena like human adult metamorphosis, as we shall see in the following papers.

## A GENERAL MODEL FOR INFORMATION IN BIOLOGICAL SYSTEMS

### Levels and Dimensions

The dimensions of space are characterized by giving degrees of freedom to a system. By thinking abstract, we can imagine these freedom-degrees of dimensions, and a new, more elastic interpretation of reality is quite conceivable to give more degrees of freedom than our well-known three-dimensional space allows. Fig. 1 gives a proposal for such an organization of the space, from three dimensions to an infinite amount of "pseudo-dimensions" (not perceptionally accessible dimensions). A spiral-fractal, that through its participation on many levels gives a big amount of curvilinear dimensions inside each other. Each of the curvilinear dimensions, have the freedom to contain its own patterns or organizations. However, these are mutually subjected to interactions from the patterns on the other levels – the curvilinear dimensions on the upper and lower levels.

Without computer implementing of the geometry, it is not clear whether the curvilinear dimensions in the spiral fractals have these interacting qualities. If this is not the case, the geometry has to be adapted. However, it seems reasonable that the same power that organizes the spiral-fractal, should also be capable of distributing all patterns evenly through the levels of the fractal, in a such way that the fractal is realized. This could be called the fractal power of the system. This power may be opposed by the fundamental, structural differences existing on the systems different levels. In this way, the organization of particles to atoms, and atoms to molecules, organelles, cells, etc….to organisms, is equivalent to each isolated level of the fractal. The elements of this, also if they have a high degree of individualism caused by information directed self-organization that acts on each level – this is called a complex epigenesis. Epigenesis is the developmental process where each successive stage of normal development is built up on the foundations created by the preceding stages of development; an embryo is built up from a zygote, a seedling from an embryo, and so on. Ontogenetic development according to epigenesis is a process of increasing complexity. This contrasts with preformationism, where the organism is already present in the gamete(s), merely growing and unfolding during development.

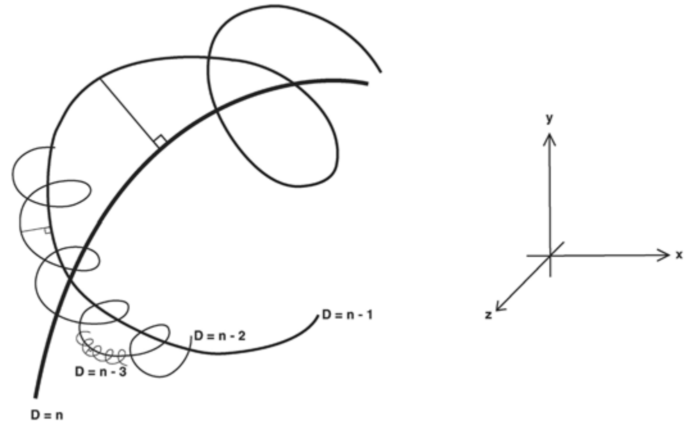

**FIGURE 1AB.** Shows a breakdown of the three dimensional space into an infinite number of curvilinear pseudodimensions. (A) Different fractal dimensions. (B) 3-D dimensions. D = Dimensions. n – 1, n – 2, n – 3 illustrates the different levels of dimensions.

It does not clearly appear what riches of patterns an expansion of the fractal geometry to 3-dimentions can involve. But, if the geometry does not involve an expansion in the amount of patterns that exceeds the 2-dimentional Mandelbrot-fractal selection, and underscore the 4-dimentional Mandelbrot-fractal selection, this 3-dimentional fractal geometry has to be changed. The work represented here may not be thought of as a complete answer on the structure of the reality and the problems of the biology, but only as an attempt to establish a completely new interdisciplinary paradigm capable of explaining a lot of unanswered questions.

## Energy, Information, and Matter

In the rational-mechanical interpretation of reality the starting point is matter represented by a particle. But in the energetic-informational interpretation of reality matter is distributed all along the spiral fractal. In this interpretation matter is not existing in a well-defined way as particles, but only as interacting organizations in the curvilinear dimensions: manifestations of energy and information. The simplest way to represent energy in the system is a consequence of destructive and constructive interference between the windings of the spiral. Destructive interference corresponds to annulment of the non-matter basic polarization, and constructive interference corresponds to an increase of the polarization (see Fig. 2). Information is found as patterns established by the storage in stable configurations or is moved as patterns between windings at the same level or up and down the dimensions. Particles may be formed by very stable configurations, in spirals that seem to be able to close itself in a circle; at a finer level they are simply complex oscillations around an average point (see Fig. 3). Systems of this type can presumably be constructed in a way, where patterns by means of interactions can be transferred between elements at the same level, and between different levels (see Fig. 4). The particles belonging to such a system will organize themselves according to their inner structure and spontaneously recreate the spiral fractal, when other forces do not hinder it (see Fig. 5). This is because of their inherent lack of an independent, particular existence and their fluid nature.

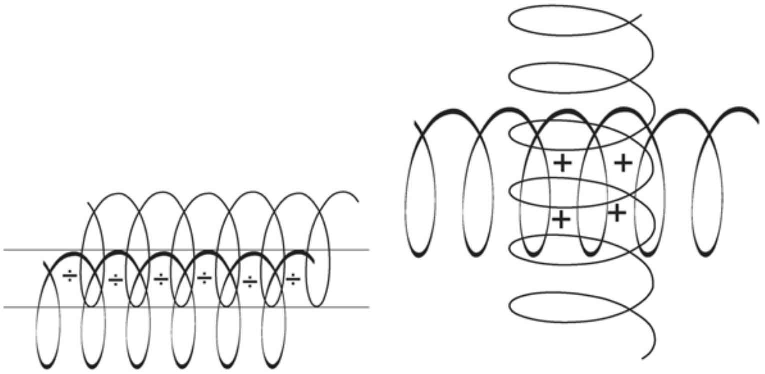

**FIGURE 2AB.** This figure illustrates the simplest way to represent energy in the fractal system is a by destructive and constructive interference between the windings of the fractal spirals. Destructive interference (minus) corresponds to annulment of the non-matter basic polarization, and constructive (plus) interference corresponds to an increase of the polarization. (A) Destructive and (B) Constructive interference.

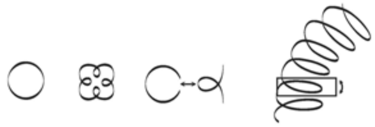

**FIGURE 3.** Particles are shown as standing waves and as stable vibrations that can be converted. An apparent particle at level n is stable oscillations at level n-1.

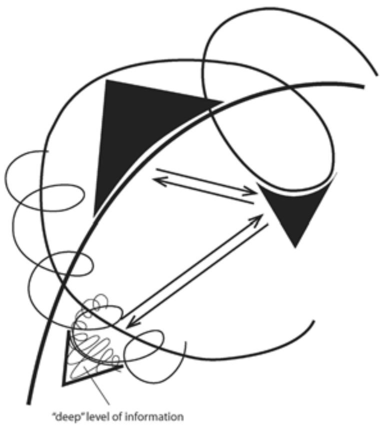

**FIGURE 4.** Illustration of information transmitting interactions. Interactions (arrows) are transferred between elements of same level, and between different levels (“the information-transferring power of the fractal”).

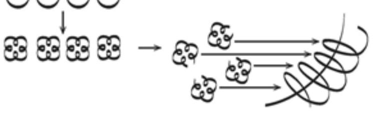

**FIGURE 5.** Self-organization of “particles” in the pattern of spiral fractals.

## Time

In the spiral-fractal time exists as dynamic patterns only. One could imagine impulses (information patterns) traveling within the fractal (around in a level or up and down through the levels) and returning with definite time intervals, giving rise to chronometers and energetic watches. We think such an energetic mechanism seems more reasonable then molecular mechanism.

## DISCUSSION

The model presented should not be thought of as an attempt to provide a definite answer to the problems of biology and the structure of reality, but as an attempt to establish a new interdisciplinary direction of research, that epistemologically seems fertile. Of course there is a long journey from this energetic-informational interpretation to a closely reasoned account of the mechanisms behind the natural forces and the information transmitting interactions. But it is very likely, that a more complex breakdown of reality can explain both the information transmitting interactions and the natural forces as specific patterns of energy and information. If this is the case, it could constitute the fundament of our complex, functional reality. And yet we as human beings possibly have to accept that the heart of reality evades description. In this case understanding the world through to the emotional interpretation of reality might be the only way to real knowledge of the world.

## CONCLUSION

Our model represents a holistic paradigm based on dynamic spiral-fractal spacious information carrying structures. The parts of the spiral can be compared to small energetic strings (presumably comparable to superstrings) able to explain the distribution of information necessary for the organization of the different levels of the organism during the morphogenesis, and also the higher levels of social reality from relations between biological individuals to small and large societies.

If the spiral fractal is really the backbone of the world, even black matter of the universe and the structure of the galaxies can be held by huge super-spirals penetrating the void of the physical universe. We speculate and dream, and hopefully this work will inspire other researchers and poets to speculate and dream as well, and one day we will have dreamt a new solution to the mysteries that have puzzled man for millenniums.

## ACKNOWLEDGMENTS

These studies were supported by grants from IMK Almene Fond. Our research in quality of life has been approved by the Copenhagen Scientific Ethical Committee under number (KF)V.100.2123/91.
